# Chronic Inflammatory Demyelinating Polyneuropathy Following Lumbar Spine Surgery in a Patient With Sarcoidosis

**DOI:** 10.7759/cureus.64578

**Published:** 2024-07-15

**Authors:** Conor Jones, Alexander P Hughes

**Affiliations:** 1 Orthopedic Surgery, Hospital for Special Surgery, New York, USA

**Keywords:** neuropathy, lumbar spine surgery, dome laminoplasty, chronic inflammatory demyelinating polyneuropathy, sarcoidosis

## Abstract

Chronic inflammatory demyelinating polyneuropathy (CIDP) is a rare relapsing-remitting autoimmune polyneuropathy that targets peripheral nerves and has been associated in the literature with sarcoidosis. The goal of this study is to report the clinical case of a 61-year-old man with sarcoidosis who developed CIDP following lumbar spine surgery. The patient presented at their clinic visit with lumbar back pain and underwent a dome laminoplasty at L2-3, L3-4, and L4-5 with no known complications. Approximately four hours postoperatively, he developed bilateral lower extremity weakness most prominent along the tibialis anterior and extensor hallucis longus (L4-S1) as well as saddle anesthesia. An MRI revealed no acute changes concerning compression. Electromyography (EMG) was performed six months postoperatively, which revealed absent F waves along the peroneal and tibial nerves as well as decreased amplitude consistent with an underlying axonal neuropathy. He was referred to a neurologist for a second opinion where a diagnosis of CIDP was made. Intravenous immune globulin treatment was initiated, and the patient felt improvement in his symptoms. This case highlights the association between sarcoidosis and CIDP and discusses the pathophysiology of the disease. In patients with sarcoidosis and weakness following lumbar surgery with a negative MRI, CIDP should be on the differential.

## Introduction

Sarcoidosis is an autoimmune inflammatory disorder that affects multiple organ systems. The incidence of sarcoidosis is estimated to range from 5 to 40 cases per 100,000 people annually. Approximately 5% of patients with sarcoidosis will have neurological involvement [1]. The presentation of sarcoid neuropathy is varied and can include cranial nerves, neurosarcoidosis, peripheral neuropathy, and sensory changes [2]. Rarely, this neuropathy presents as chronic inflammatory demyelinating polyneuropathy (CIDP) [3-5], an immune-mediated polyneuropathy that targets peripheral nerves. CIDP has an estimated prevalence of 1 to 2 per 100,000 adults, with the hallmark of the condition being rapidly progressive motor symmetrical motor loss that lasts greater than two months [6]. While an association between sarcoidosis and CIDP has been shown in the literature, the trigger of CIDP remains unknown. We herein report a case of a patient with sarcoidosis who developed CIDP following lumbar decompression surgery.

## Case presentation

A 61-year-old male with a history of sarcoidosis presented at his clinic visit with 15 years of chronic lumbar pain. His symptoms included pins and needles in the bilateral posterolateral thighs that worsened with standing. On motor exam, his strength was 5/5 throughout and sensation was intact. He had tried epidural steroid injections, physical therapy, and nonsteroidal anti-inflammatory drugs with limited response. A completed MRI showed spinal stenosis at L2-3, L3-4, and L4-5.

The patient underwent a left-sided micro-laminectomy and dome laminoplasty at L2-3, L3-4, and L4-5, along with a left microdiscectomy at L4-5 in the prone position. There were no intraoperative complications.

**Figure 1:**
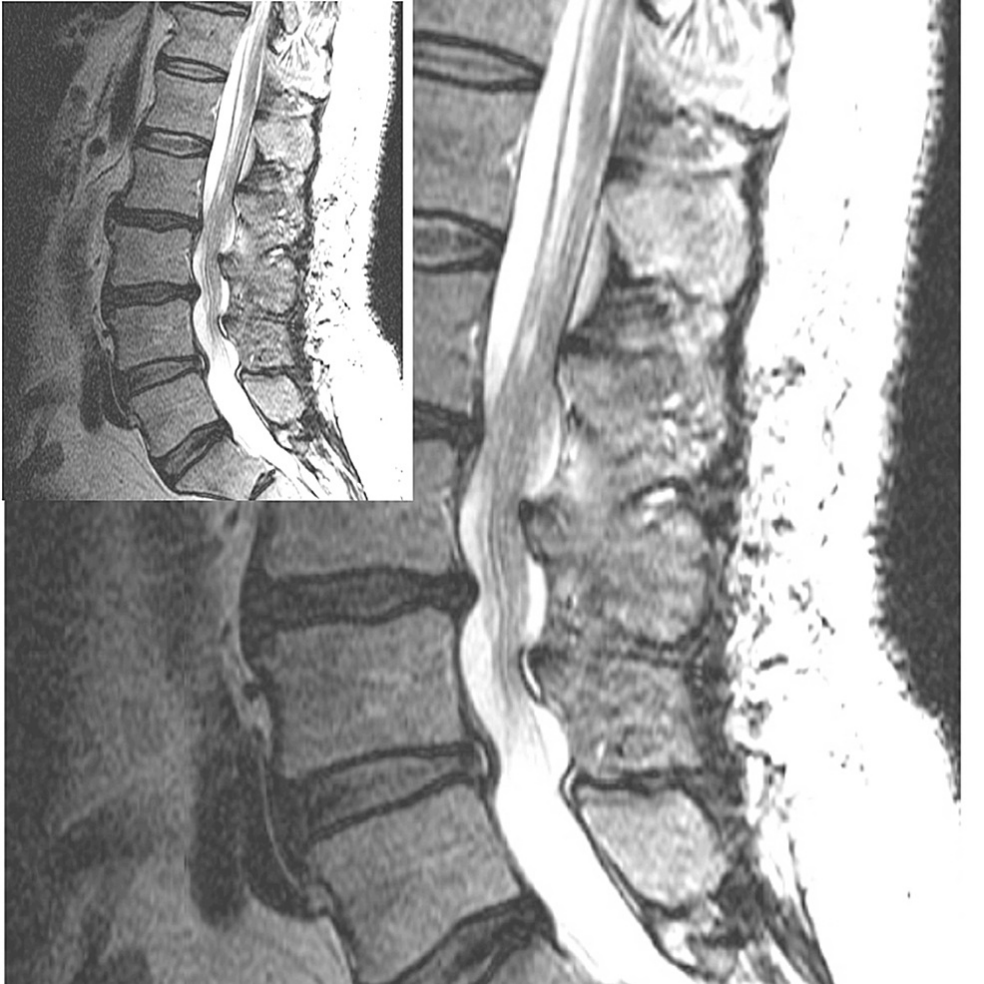
Postoperative T2-weighted MRI of the lumbar spine demonstrating no compressive elements (hematoma or seroma) on the spinal cord.

Postoperatively, the patient was found to have acute bilateral lower extremity weakness, saddle anesthesia, and incontinence. An MRI with gadolinium contrast of the thoracolumbar spine was performed and showed no spinal cord edema, no substantial canal or neural foraminal stenosis, and no evidence of a compressive hematoma (Figure 1). The patient was started on a course of dexamethasone. Motor and voiding symptoms slowly improved, and the patient was discharged to rehab 20 days postoperatively. At the time of discharge, the right lower extremity strength of the tibialis anterior (TA) and extensor hallucis longus (EHL) was 1/5, improved from 0/5 immediately postoperatively.

Three months following surgery, the patient continued to have proprioceptive and sensory deficits in his bilateral lower extremities, neuropathic pain, and weakness in the right lower extremity. One year postoperatively, the patient had limited improvement in his symptoms with right TA and EHL strength 3/5 and 2/5, respectively. Electromyography (EMG) was performed six months postoperatively, which showed absent F waves bilaterally along the peroneal and tibial nerves as well as decreased amplitudes consistent with an underlying axonal polyneuropathy. Given the patient’s limited improvement over one year postoperatively, EMG suggesting an underlying axonal polyneuropathy, and negative MRI findings for compression, the patient was referred to a separate neurologist for a second opinion. There, a diagnosis of CIDP was made. The patient was started on a course of intravenous immunoglobulin (IVIG) and experienced an improvement in his symptoms.

## Discussion

CIDP is a relapsing-remitting autoimmune disorder that presents with sensory deficits and weakness [6]. The pathogenesis of CIDP is thought to stem from the process of molecular mimicry, in which immune cells become autoreactive and sensitized to the host’s neural cells after exposure to a triggering antigen. This is an area of ongoing investigation, as viruses, autoimmune diseases, and certain cancers have been shown to precede CIDP. Once T-cells become autoreactive and lose self-tolerance, they cross the blood-nerve barrier and release inflammatory cytokines, such as tumor necrosis factor-alpha (TNF-alpha), interferon-gamma, and interleukin-2. This process activates resident macrophages, resulting in phagocytic activity and the release of neurotoxic molecules, targeting myelin and Schwann cells and inducing apoptosis. While CIDP is a demyelinating pathology, it also adversely affects neural axons. The long-term prognosis of CIDP is largely dependent on the amount of axonal loss [6].

In patients with CIDP, the differential diagnosis is broad and can include direct trauma in the operative setting, postoperative compressive hematoma, acute inflammatory demyelinating polyneuropathy (AIDP), metabolic neuropathy, and toxic neuropathies [6]. Diagnosis of CIDP is primarily one of exclusion with insights from clinical findings, EMGs, lumbar punctures, and nerve biopsies when available. Symptoms include motor and sensory dysfunction of at least one limb for a period of greater than two months. While the two-month duration is a clear indicator of CIDP, this duration is a problematic criterion in the acute postoperative setting. There are varying EMG criteria, but common findings described in the Koski criteria include abnormal F waves, decreased amplitudes, normal compound muscle action potential, and decreased conduction velocities [7-9]. Our patient’s absent F waves and decreased amplitudes are consistent with typical CIDP EMG findings. Lumbar punctures performed in patients with CIDP demonstrate elevated protein levels and white cell counts <10/mm^3^. Nerve biopsies, although not required for diagnosis, show evidence of demyelination.

Sarcoidosis has been associated with CIDP. A review of 57 patients with sarcoid neuropathy detailed five cases of CIDP [3]. A case by Ducray et al. reported on a patient who presented with CIDP preceding the development of sarcoid symptoms [4]. Separately, a report by Mansour et al. described a patient with known sarcoidosis who developed CIDP [5]. The pathogenesis of sarcoidosis with CIDP is uncertain. One theory is perivascular granulomas may lead to nerve ischemia and demyelination [10]. Indeed, Burns et al. described CIDP in a patient with sarcoidosis where nerve biopsies demonstrated a non-caseating granuloma very close to a vessel [11]. In a separate work examining the relationship between sarcoidosis and neuropathy, Said et al. hypothesized that these granulomas in sarcoidosis secrete inflammatory cytokines from memory T-cells within the granuloma. When the granuloma invades the endoneural blood vessels, these inflammatory cytokines can activate macrophages and damage the vessel wall, leading the fibrinoid necrosis [12]. It is theorized that a combination of ischemia and inflammatory cytokines, leading to macrophage activation, results in demyelination and eventual axonal loss [10,12].

Although sarcoidosis has been associated with CIDP, there are no case reports in the literature that describe spinal surgery as the triggering event for CIDP. However, cases are detailing AIDP, which has a similar pathogenesis but has symptoms for less than two months, after spinal surgeries. One report described a pair of patients who presented with AIDP within hours after lumbar laminectomies. Both cases had lower extremity weakness with no MRI changes and responded to IVIG treatment [13]. A separate case detailed an incidence of AIDP following a lumbar laminectomy that also improved with IVIG [14]. It has been theorized that direct trauma to the nerve root releases antigens that sensitize immune cells and initiate an autoimmune sequence that leads to demyelinating neuropathy in susceptible patients [14,15]. In addition to this antigen release, surgery has been thought to alter the balance of the immune system and result in a transient immunosuppression [16]. This combination of immunosuppression with antigen release is thought to cause this global neuropathy following spinal surgery. In patients with sarcoidosis and perivascular granulomas, an antigenic release following nerve root stimulation may lead to prolonged memory T-cell activation within these granulomas, resulting in CIDP.

## Conclusions

In this study, we report on a patient with sarcoidosis who developed CIDP following a lumbar decompression. Previous reports have demonstrated that lumbar surgery may act as a potential nidus for the release of antigens, triggering an autoimmune demyelinating attack. Patients with sarcoidosis are at an increased risk for demyelinating pathologies secondary to perivascular granulomas and sustained release of cytokines within the endoneural space. In cases of postoperative weakness and no MRI changes following lumbar surgery in a patient with sarcoidosis, we recommend that surgeons have CIDP on their differential.
